# 9-(2,6-Dimethyl­phen­oxy­carbon­yl)-10-methyl­acridinium trifluoro­methane­sulfonate

**DOI:** 10.1107/S1600536810041449

**Published:** 2010-10-23

**Authors:** Damian Trzybiński, Karol Krzymiński, Jerzy Błażejowski

**Affiliations:** a Faculty of Chemistry, University of Gdańsk, J. Sobieskiego 18, 80-952 Gdańsk, Poland

## Abstract

In the crystal structure of the title compound, C_23_H_20_NO_2_^+^·CF_3_SO_3_^−^, adjacent cations are linked through a network of C—H⋯π and π–π inter­actions, and neighboring cations and anions *via* C—H⋯O inter­actions. The acridine and benzene ring systems are oriented at a dihedral angle of 31.4 (1)°. The carboxyl group is twisted at an angle of 66.3 (1)° relative to the acridine skeleton. The mean planes of the adjacent acridine moieties are parallel in the crystal structure.

## Related literature

For general background to the chemiluminogenic properties of 9-phen­oxy­carbonyl-10-methyl­acridinium trifluoro­meth­ane­sulfonates, see: Brown *et al.* (2009 ▶); Natrajan *et al.* (2010 ▶). For related structures, see: Krzymiński *et al.* (2009 ▶); Niziołek *et al.* (2009 ▶). For inter­molecular inter­actions, see: Dorn *et al.* (2005 ▶); Hunter *et al.* (2001 ▶); Novoa *et al.* (2006 ▶); Takahashi *et al.* (2001 ▶). For the synthesis, see: Sato (1996 ▶); Niziołek *et al.* (2009 ▶).
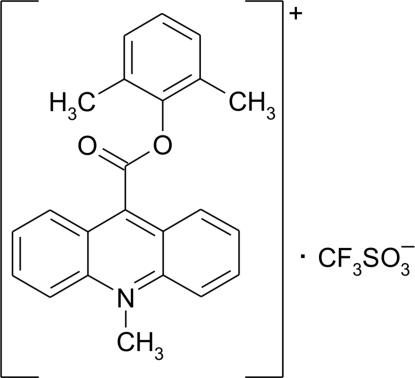


## Experimental

### 

#### Crystal data

C_23_H_20_NO_2_^+^·CF_3_SO_3_^−^
*M* *_r_* = 491.48
Triclinic, 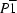

*a* = 9.5841 (4) Å
*b* = 11.2491 (6) Å
*c* = 12.1738 (3) Å
α = 106.080 (3)°
β = 101.890 (3)°
γ = 110.755 (4)°
*V* = 1109.66 (8) Å^3^
*Z* = 2
Mo *K*α radiation
μ = 0.21 mm^−1^
*T* = 295 K
0.58 × 0.18 × 0.05 mm

#### Data collection

Oxford Diffraction Gemini R Ultra Ruby CCD diffractometer
9670 measured reflections
3922 independent reflections
3124 reflections with *I* > 2σ(*I*)
*R*_int_ = 0.016

#### Refinement

*R*[*F* ^2^ > 2σ(*F* ^2^)] = 0.042
*wR*(*F* ^2^) = 0.125
*S* = 1.09
3922 reflections
321 parameters
6 restraints
H atoms treated by a mixture of independent and constrained refinement
Δρ_max_ = 0.33 e Å^−3^
Δρ_min_ = −0.27 e Å^−3^

### 

Data collection: *CrysAlis CCD* (Oxford Diffraction, 2008 ▶); cell refinement: *CrysAlis RED* (Oxford Diffraction, 2008 ▶); data reduction: *CrysAlis RED*; program(s) used to solve structure: *SHELXS97* (Sheldrick, 2008 ▶); program(s) used to refine structure: *SHELXL97* (Sheldrick, 2008 ▶); molecular graphics: *ORTEP-3* (Farrugia, 1997 ▶); software used to prepare material for publication: *SHELXL97* and *PLATON* (Spek, 2009 ▶).

## Supplementary Material

Crystal structure: contains datablocks global, I. DOI: 10.1107/S1600536810041449/ng5042sup1.cif

Structure factors: contains datablocks I. DOI: 10.1107/S1600536810041449/ng5042Isup2.hkl

Additional supplementary materials:  crystallographic information; 3D view; checkCIF report

## Figures and Tables

**Table 1:** Hydrogen-bond geometry (Å, °) *Cg*4 is the centroid of the C18–C23 ring.

*D*—H⋯*A*	*D*—H	H⋯*A*	*D*⋯*A*	*D*—H⋯*A*
C2—H2⋯O28^i^	0.93	2.51	3.224 (3)	134
C25—H25*B*⋯O30^ii^	0.96	2.57	3.525 (3)	176
C26—H26*A*⋯*Cg*4^iii^	0.96 (3)	2.86 (2)	3.774 (3)	158 (3)
C26—H26*B*⋯O29^iii^	0.96 (3)	2.56 (3)	3.369 (4)	142 (2)

Symmetry codes: (i) 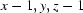; (ii) 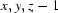; (iii) 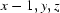.

**Table 2:** π–π inter­actions (Å,°) *Cg*1, *Cg*2 and *Cg*3 are the centroids of the C9/N10/C11–C14, C1–C4/C11/C12 and C5–C8/C13/C14 rings, respectively. *CgI*⋯*CgJ* is the distance between ring centroids. The dihedral angle is that between the planes of the rings *I* and *J. CgI*_Perp is the perpendicular distance of *CgI* from ring *J. CgI*_Offset is the distance between *CgI* and perpendicular projection of *CgJ* on ring *I*.

*I*	*J*	*CgI*⋯*CgJ*	Dihedral angle	*CgI*_Perp	*CgI*_Offset
1	3^v^	3.502 (2)	2.71 (10)	3.473 (1)	0.445 (1)
2	3^v^	3.977 (2)	6.38 (11)	3.286 (1)	2.240 (1)
3	1^v^	3.502 (2)	2.71 (10)	3.470 (1)	0.480 (1)
3	2^v^	3.977 (2)	6.38 (11)	3.503 (1)	1.883 (1)

Symmetry code: (v) 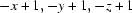.

**Fig. 1.**
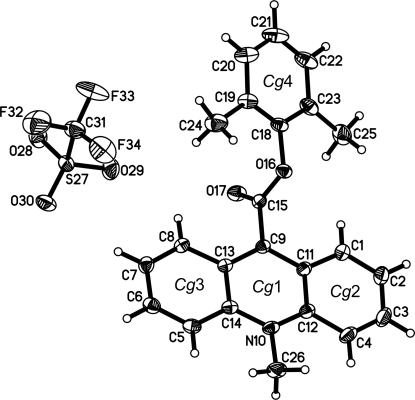
The molecular structure of the title compound showing the atom-labeling scheme. Displacement ellipsoids are drawn at the 25% probability level and H atoms are shown as small spheres of arbitrary radius. Cg1, Cg2, Cg3 and Cg4 denote the ring centroids.

**Fig. 2.**
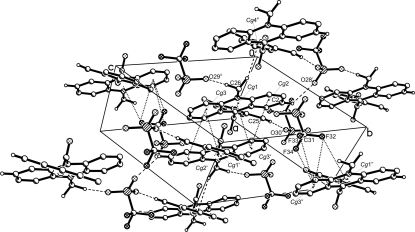
The arrangement of the ions in the crystal structure. The C–H···O and C–H···π interactions are represented by dashed lines, the C–F···π and π–π contacts by dotted lines. H atoms not involved in interactions have been omitted. [Symmetry codes: (i) x – 1, y, z – 1; (ii) x, y, z – 1; (iii) x – 1, y, z; (iv) –x + 1, –y, –z + 1; (v) –x + 1, –y + 1, –z + 1.]

## supplementary crystallographic information

### Comment

Chemiluminescent indicators or the chemiluminogenic fragments of chemiluminescent labels based on 9-(phenoxycarbonyl)-10-methylacridinium salts are widely used in assays of biologically and environmentally important entities such as antigens, antibodies, enzymes or DNA fragments (Brown *et al.*, 2009; Natrajan *et al.*, 2010). The efficiency of chemiluminescence – crucial for analytical applications – is affected by the structure of the phenyl fragment. We thus undertook investigations into 9-(phenoxycarbonyl)-10-methylacridinium salts variously substituted at the benzene ring. Here we present the structure of 9-(2,6-dimethylphenoxycarbonyl)-10-methylacridinium trifluoromethanesulfonate.

In the cation of the title compound (Fig. 1), the bond lengths and angles characterizing the geometry of the acridinium moiety are typical of acridine-based derivatives (Krzymiński *et al.*, 2009; Niziołek *et al.*, 2009). With respective average deviations from planarity of 0.0629 (3) Å and 0.0046 (3) Å, the acridine and benzene ring systems are oriented at a dihedral angle of 31.4 (1)°. The carboxyl group is twisted at an angle of 66.3 (1)° relative to the acridine skeleton. The mean planes of the adjacent acridine moieties are parallel (remain at an angle 0.0 (1)°) in the lattice.

In the crystal structure, the inversely oriented cations are linked through a network of C–H···π (Table 1, Fig. 2) and π–π (Table 3, Fig. 2) interactions, the adjacent cations and anions via C–H···O (Table 1, Fig. 2) and C–F···π (Table 2, Fig. 2) interactions. The C–H···O (Novoa *et al.* 2006) interactions are of the hydrogen bond type. The C–H···π (Takahashi *et al.* 2001) interactions should be of an attractive nature, like C–F···π (Dorn *et al.*, 2005) and the π–π (Hunter *et al.*, 2001) interactions. The crystal structure is stabilized by a network of these short-range specific interactions and by long-range electrostatic interactions between ions.

### Experimental

2,6-Dimethylphenylacridine-9-carboxylate was synthesized first in the reaction of 9-(chlorocarbonyl)acridine (obtained by treating acridine-9-carboxylic acid with a tenfold molar excess of thionyl chloride) with 2,6-dimethylphenol in anhydrous dichloromethane in the presence of *N*,*N*-diethylethanamine and a catalytic amount of *N*,*N*-dimethyl-4-pyridinamine (room temperature, 15h) (Sato, 1996). The ester thereby obtained, purified chromatographically (SiO_2_, cyclohexane/ethyl acetate, 1/1 v/v), was quaternarized with a fivefold molar excess of methyl trifluoromethanesulfonate dissolved in anhydrous dichloromethane. The crude 9-(2,6-dimethylphenoxycarbonyl)-10-methylacridinium trifluoromethanesulfonate was dissolved in a small amount of ethanol, filtered and precipitated with 20 v/v excess of diethyl ether. Yellow crystals suitable for X-ray investigations were grown from absolute ethanol solution (m.p. 552–555 K).

### Refinement

The H26A, H26B and H26C atoms were located on a Fourier-difference map, restrained by DFIX command 0.960 for C–H distance and by DFIX 1.568 for H···H distance, and refined as riding with *U*_iso_(H) = 1.5*U*_eq_(C). All other H atoms were positioned geometrically, with C—H = 0.93 Å and 0.96 Å for the aromatic and methyl H atoms, respectively, and constrained to ride on their parent atoms with *U*_iso_(H) = *xU*_eq_(C), where *x* = 1.2 for the aromatic and *x* = 1.5 for the methyl H atoms.

### Crystal data

**Table:**

C_23_H_20_NO_2_^+^·CF_3_SO_3_^−^	*Z* = 2
*M**_r_* = 491.48	*F*(000) = 508
Triclinic, *P*1	*D*_x_ = 1.471 Mg m^−^^3^
Hall symbol: -P 1	Mo *K*α radiation, λ = 0.71073 Å
*a* = 9.5841 (4) Å	Cell parameters from 66666 reflections
*b* = 11.2491 (6) Å	θ = 3.0–29.1°
*c* = 12.1738 (3) Å	µ = 0.21 mm^−^^1^
α = 106.080 (3)°	*T* = 295 K
β = 101.890 (3)°	Prism, light-yellow
γ = 110.755 (4)°	0.58 × 0.18 × 0.05 mm
*V* = 1109.66 (8) Å^3^	

### Data collection

**Table:**

Oxford Diffraction Gemini R Ultra Ruby CCD diffractometer	3124 reflections with *I* > 2σ(*I*)
Radiation source: Enhanced (Mo) X-ray Source	*R*_int_ = 0.016
graphite	θ_max_ = 25.1°, θ_min_ = 3.1°
Detector resolution: 10.4002 pixels mm^-1^	*h* = −11→11
ω scans	*k* = −13→10
9670 measured reflections	*l* = −14→14
3922 independent reflections	

### Refinement

**Table:**

Refinement on *F*^2^	Primary atom site location: structure-invariant direct methods
Least-squares matrix: full	Secondary atom site location: difference Fourier map
*R*[*F*^2^ > 2σ(*F*^2^)] = 0.042	Hydrogen site location: inferred from neighbouring sites
*wR*(*F*^2^) = 0.125	H atoms treated by a mixture of independent and constrained refinement
*S* = 1.09	*w* = 1/[σ^2^(*F*_o_^2^) + (0.0673*P*)^2^ + 0.2264*P*] where *P* = (*F*_o_^2^ + 2*F*_c_^2^)/3
3922 reflections	(Δ/σ)_max_ = 0.001
321 parameters	Δρ_max_ = 0.33 e Å^−^^3^
6 restraints	Δρ_min_ = −0.27 e Å^−^^3^

### Special details

**Table:**

Geometry. All esds (except the esd in the dihedral angle between two l.s. planes) are estimated using the full covariance matrix. The cell esds are taken into account individually in the estimation of esds in distances, angles and torsion angles; correlations between esds in cell parameters are only used when they are defined by crystal symmetry. An approximate (isotropic) treatment of cell esds is used for estimating esds involving l.s. planes.

### Fractional atomic coordinates and isotropic or equivalent isotropic displacement parameters (Å^2^)

**Table:**

	*x*	*y*	*z*	*U*_iso_*/*U*_eq_	
C1	0.3818 (3)	0.3638 (2)	0.13066 (19)	0.0649 (6)	
H1	0.4654	0.3528	0.1091	0.078*	
C2	0.2838 (3)	0.3952 (3)	0.0588 (2)	0.0777 (7)	
H2	0.2996	0.4048	−0.0118	0.093*	
C3	0.1595 (3)	0.4130 (3)	0.0913 (2)	0.0787 (8)	
H3	0.0932	0.4350	0.0419	0.094*	
C4	0.1322 (3)	0.3992 (2)	0.1928 (2)	0.0687 (6)	
H4	0.0489	0.4130	0.2125	0.082*	
C5	0.2865 (2)	0.3196 (2)	0.56175 (19)	0.0545 (5)	
H5	0.1991	0.3261	0.5797	0.065*	
C6	0.3931 (3)	0.3026 (2)	0.6409 (2)	0.0592 (5)	
H6	0.3771	0.2975	0.7126	0.071*	
C7	0.5267 (2)	0.2924 (2)	0.61744 (18)	0.0557 (5)	
H7	0.5987	0.2820	0.6737	0.067*	
C8	0.5505 (2)	0.29779 (19)	0.51298 (17)	0.0479 (4)	
H8	0.6386	0.2898	0.4975	0.057*	
C9	0.46128 (19)	0.32004 (18)	0.31651 (16)	0.0424 (4)	
N10	0.20515 (16)	0.34795 (16)	0.37176 (15)	0.0499 (4)	
C11	0.3594 (2)	0.34746 (19)	0.23794 (16)	0.0474 (4)	
C12	0.2299 (2)	0.36386 (19)	0.26929 (17)	0.0505 (5)	
C13	0.44292 (19)	0.31544 (17)	0.42602 (15)	0.0411 (4)	
C14	0.30806 (19)	0.32732 (18)	0.45257 (17)	0.0448 (4)	
C15	0.5963 (2)	0.29831 (19)	0.28346 (16)	0.0436 (4)	
O16	0.53926 (14)	0.18238 (13)	0.18486 (11)	0.0503 (3)	
O17	0.73309 (15)	0.37184 (15)	0.33881 (13)	0.0625 (4)	
C18	0.6473 (2)	0.1321 (2)	0.15004 (17)	0.0514 (5)	
C19	0.6730 (3)	0.0416 (2)	0.1981 (2)	0.0641 (6)	
C20	0.7664 (3)	−0.0180 (3)	0.1546 (3)	0.0849 (8)	
H20	0.7870	−0.0800	0.1844	0.102*	
C21	0.8276 (3)	0.0133 (3)	0.0693 (3)	0.0922 (9)	
H21	0.8888	−0.0280	0.0412	0.111*	
C22	0.8000 (3)	0.1047 (3)	0.0246 (2)	0.0813 (8)	
H22	0.8428	0.1245	−0.0338	0.098*	
C23	0.7089 (2)	0.1689 (2)	0.06460 (19)	0.0631 (6)	
C24	0.6045 (4)	0.0075 (3)	0.2916 (3)	0.0923 (9)	
H24A	0.4922	−0.0213	0.2627	0.138*	
H24B	0.6527	0.0874	0.3658	0.138*	
H24C	0.6248	−0.0654	0.3061	0.138*	
C25	0.6814 (3)	0.2718 (3)	0.0184 (2)	0.0835 (8)	
H25A	0.5697	0.2402	−0.0195	0.125*	
H25B	0.7338	0.2826	−0.0399	0.125*	
H25C	0.7232	0.3586	0.0850	0.125*	
C26	0.0613 (3)	0.3550 (3)	0.3959 (3)	0.0779 (8)	
H26A	−0.027 (3)	0.305 (3)	0.3197 (17)	0.113 (10)*	
H26B	0.034 (3)	0.307 (3)	0.448 (2)	0.106 (11)*	
H26C	0.069 (5)	0.4454 (19)	0.431 (3)	0.184 (19)*	
S27	0.95913 (6)	0.27457 (6)	0.72251 (5)	0.05989 (19)	
O28	1.1219 (2)	0.3034 (3)	0.77069 (16)	0.1008 (7)	
O29	0.9225 (2)	0.3134 (2)	0.62202 (16)	0.0875 (5)	
O30	0.8851 (2)	0.3042 (2)	0.81008 (15)	0.0879 (6)	
C31	0.8618 (4)	0.0892 (3)	0.6536 (3)	0.0856 (8)	
F32	0.8764 (3)	0.0335 (2)	0.7353 (3)	0.1501 (9)	
F33	0.9169 (3)	0.0417 (2)	0.5713 (2)	0.1554 (10)	
F34	0.7072 (2)	0.0441 (2)	0.5986 (2)	0.1331 (8)	

### Atomic displacement parameters (Å^2^)

**Table:**

	*U*^11^	*U*^22^	*U*^33^	*U*^12^	*U*^13^	*U*^23^
C1	0.0770 (14)	0.0765 (15)	0.0556 (12)	0.0464 (13)	0.0212 (10)	0.0297 (11)
C2	0.1007 (19)	0.0832 (18)	0.0582 (13)	0.0549 (16)	0.0142 (13)	0.0298 (12)
C3	0.0827 (17)	0.0773 (17)	0.0680 (15)	0.0458 (14)	−0.0039 (13)	0.0235 (13)
C4	0.0540 (12)	0.0665 (14)	0.0776 (15)	0.0362 (11)	0.0030 (11)	0.0176 (12)
C5	0.0524 (11)	0.0474 (11)	0.0663 (12)	0.0206 (9)	0.0297 (10)	0.0199 (9)
C6	0.0715 (13)	0.0539 (12)	0.0596 (11)	0.0266 (10)	0.0331 (10)	0.0252 (10)
C7	0.0611 (12)	0.0542 (12)	0.0549 (11)	0.0254 (10)	0.0182 (9)	0.0261 (9)
C8	0.0437 (9)	0.0490 (11)	0.0550 (10)	0.0226 (8)	0.0171 (8)	0.0220 (9)
C9	0.0369 (8)	0.0385 (9)	0.0483 (9)	0.0161 (7)	0.0119 (7)	0.0140 (8)
N10	0.0350 (7)	0.0455 (9)	0.0609 (9)	0.0192 (7)	0.0114 (7)	0.0099 (7)
C11	0.0448 (9)	0.0433 (10)	0.0497 (10)	0.0214 (8)	0.0091 (8)	0.0139 (8)
C12	0.0421 (9)	0.0422 (10)	0.0541 (11)	0.0186 (8)	0.0046 (8)	0.0084 (8)
C13	0.0355 (8)	0.0346 (9)	0.0484 (9)	0.0134 (7)	0.0128 (7)	0.0130 (7)
C14	0.0373 (9)	0.0344 (9)	0.0546 (10)	0.0125 (7)	0.0149 (8)	0.0105 (8)
C15	0.0425 (10)	0.0471 (10)	0.0475 (9)	0.0223 (8)	0.0170 (8)	0.0222 (8)
O16	0.0422 (6)	0.0538 (8)	0.0512 (7)	0.0208 (6)	0.0188 (5)	0.0130 (6)
O17	0.0392 (7)	0.0589 (9)	0.0731 (9)	0.0175 (6)	0.0162 (6)	0.0095 (7)
C18	0.0414 (9)	0.0491 (11)	0.0562 (11)	0.0166 (8)	0.0223 (8)	0.0097 (9)
C19	0.0627 (12)	0.0548 (13)	0.0801 (14)	0.0277 (11)	0.0356 (11)	0.0225 (11)
C20	0.0797 (16)	0.0653 (16)	0.122 (2)	0.0416 (14)	0.0483 (16)	0.0305 (15)
C21	0.0762 (16)	0.0717 (17)	0.129 (2)	0.0345 (14)	0.0622 (17)	0.0164 (17)
C22	0.0696 (15)	0.0780 (18)	0.0849 (16)	0.0207 (13)	0.0505 (13)	0.0128 (14)
C23	0.0526 (11)	0.0643 (14)	0.0577 (11)	0.0143 (10)	0.0260 (9)	0.0121 (10)
C24	0.125 (2)	0.0864 (19)	0.118 (2)	0.0658 (18)	0.0734 (19)	0.0631 (17)
C25	0.0869 (17)	0.106 (2)	0.0748 (15)	0.0398 (16)	0.0463 (13)	0.0480 (15)
C26	0.0458 (12)	0.101 (2)	0.0848 (17)	0.0402 (13)	0.0235 (12)	0.0217 (16)
S27	0.0655 (3)	0.0737 (4)	0.0501 (3)	0.0355 (3)	0.0266 (2)	0.0255 (3)
O28	0.0647 (10)	0.1509 (19)	0.0711 (11)	0.0372 (11)	0.0187 (8)	0.0367 (12)
O29	0.1033 (13)	0.1112 (14)	0.0781 (11)	0.0551 (12)	0.0399 (10)	0.0610 (11)
O30	0.1185 (15)	0.1062 (14)	0.0730 (10)	0.0705 (12)	0.0590 (10)	0.0348 (10)
C31	0.101 (2)	0.091 (2)	0.0888 (18)	0.0516 (17)	0.0548 (16)	0.0386 (16)
F32	0.212 (3)	0.1266 (17)	0.191 (2)	0.1025 (18)	0.106 (2)	0.1062 (17)
F33	0.187 (2)	0.1174 (16)	0.164 (2)	0.0741 (16)	0.1115 (18)	0.0081 (14)
F34	0.0897 (13)	0.1208 (16)	0.1312 (15)	0.0049 (11)	0.0360 (11)	0.0222 (12)

### Geometric parameters (Å, °)

**Table:**

C1—C2	1.357 (3)	O16—C18	1.426 (2)
C1—C11	1.417 (3)	C18—C19	1.374 (3)
C1—H1	0.9300	C18—C23	1.385 (3)
C2—C3	1.392 (4)	C19—C20	1.398 (3)
C2—H2	0.9300	C19—C24	1.498 (3)
C3—C4	1.352 (4)	C20—C21	1.361 (4)
C3—H3	0.9300	C20—H20	0.9300
C4—C12	1.419 (3)	C21—C22	1.366 (4)
C4—H4	0.9300	C21—H21	0.9300
C5—C6	1.357 (3)	C22—C23	1.396 (3)
C5—C14	1.408 (3)	C22—H22	0.9300
C5—H5	0.9300	C23—C25	1.497 (4)
C6—C7	1.404 (3)	C24—H24A	0.9600
C6—H6	0.9300	C24—H24B	0.9600
C7—C8	1.351 (3)	C24—H24C	0.9600
C7—H7	0.9300	C25—H25A	0.9600
C8—C13	1.427 (3)	C25—H25B	0.9600
C8—H8	0.9300	C25—H25C	0.9600
C9—C13	1.393 (3)	C26—H26A	0.971 (16)
C9—C11	1.398 (3)	C26—H26B	0.966 (16)
C9—C15	1.510 (2)	C26—H26C	0.956 (17)
N10—C12	1.364 (3)	S27—O30	1.4262 (17)
N10—C14	1.372 (2)	S27—O29	1.4274 (17)
N10—C26	1.492 (3)	S27—O28	1.4290 (19)
C11—C12	1.428 (3)	S27—C31	1.803 (3)
C13—C14	1.436 (2)	C31—F33	1.301 (3)
C15—O17	1.190 (2)	C31—F32	1.323 (4)
C15—O16	1.341 (2)	C31—F34	1.331 (3)
			
C2—C1—C11	121.3 (2)	C19—C18—O16	116.51 (17)
C2—C1—H1	119.3	C23—C18—O16	118.52 (19)
C11—C1—H1	119.3	C18—C19—C20	116.4 (2)
C1—C2—C3	119.5 (2)	C18—C19—C24	122.2 (2)
C1—C2—H2	120.3	C20—C19—C24	121.5 (2)
C3—C2—H2	120.3	C21—C20—C19	121.0 (3)
C4—C3—C2	122.0 (2)	C21—C20—H20	119.5
C4—C3—H3	119.0	C19—C20—H20	119.5
C2—C3—H3	119.0	C20—C21—C22	120.7 (2)
C3—C4—C12	120.4 (2)	C20—C21—H21	119.6
C3—C4—H4	119.8	C22—C21—H21	119.6
C12—C4—H4	119.8	C21—C22—C23	121.3 (2)
C6—C5—C14	120.13 (18)	C21—C22—H22	119.3
C6—C5—H5	119.9	C23—C22—H22	119.3
C14—C5—H5	119.9	C18—C23—C22	115.8 (2)
C5—C6—C7	121.8 (2)	C18—C23—C25	122.5 (2)
C5—C6—H6	119.1	C22—C23—C25	121.7 (2)
C7—C6—H6	119.1	C19—C24—H24A	109.5
C8—C7—C6	119.85 (19)	C19—C24—H24B	109.5
C8—C7—H7	120.1	H24A—C24—H24B	109.5
C6—C7—H7	120.1	C19—C24—H24C	109.5
C7—C8—C13	121.10 (18)	H24A—C24—H24C	109.5
C7—C8—H8	119.4	H24B—C24—H24C	109.5
C13—C8—H8	119.4	C23—C25—H25A	109.5
C13—C9—C11	121.32 (16)	C23—C25—H25B	109.5
C13—C9—C15	119.28 (15)	H25A—C25—H25B	109.5
C11—C9—C15	119.39 (17)	C23—C25—H25C	109.5
C12—N10—C14	122.34 (15)	H25A—C25—H25C	109.5
C12—N10—C26	118.00 (18)	H25B—C25—H25C	109.5
C14—N10—C26	119.65 (19)	N10—C26—H26A	108.1 (18)
C9—C11—C1	122.93 (18)	N10—C26—H26B	109.5 (19)
C9—C11—C12	118.46 (18)	H26A—C26—H26B	106.0 (18)
C1—C11—C12	118.58 (18)	N10—C26—H26C	116 (3)
N10—C12—C4	122.02 (19)	H26A—C26—H26C	109 (2)
N10—C12—C11	119.78 (17)	H26B—C26—H26C	108 (2)
C4—C12—C11	118.2 (2)	O30—S27—O29	115.49 (12)
C9—C13—C8	123.28 (16)	O30—S27—O28	115.69 (11)
C9—C13—C14	118.62 (16)	O29—S27—O28	114.61 (12)
C8—C13—C14	118.10 (17)	O30—S27—C31	102.71 (12)
N10—C14—C5	121.83 (17)	O29—S27—C31	102.86 (13)
N10—C14—C13	119.14 (17)	O28—S27—C31	102.72 (15)
C5—C14—C13	119.02 (17)	F33—C31—F32	108.6 (3)
O17—C15—O16	125.17 (17)	F33—C31—F34	106.8 (3)
O17—C15—C9	124.77 (17)	F32—C31—F34	106.9 (3)
O16—C15—C9	110.03 (14)	F33—C31—S27	111.8 (2)
C15—O16—C18	118.64 (14)	F32—C31—S27	111.7 (2)
C19—C18—C23	124.78 (19)	F34—C31—S27	110.7 (2)
			
C11—C1—C2—C3	0.6 (4)	C8—C13—C14—N10	178.32 (15)
C1—C2—C3—C4	−0.4 (4)	C9—C13—C14—C5	178.53 (16)
C2—C3—C4—C12	−0.9 (4)	C8—C13—C14—C5	−0.7 (2)
C14—C5—C6—C7	0.1 (3)	C13—C9—C15—O17	−63.4 (3)
C5—C6—C7—C8	−0.9 (3)	C11—C9—C15—O17	115.1 (2)
C6—C7—C8—C13	0.8 (3)	C13—C9—C15—O16	114.90 (17)
C13—C9—C11—C1	174.81 (18)	C11—C9—C15—O16	−66.6 (2)
C15—C9—C11—C1	−3.7 (3)	O17—C15—O16—C18	7.9 (3)
C13—C9—C11—C12	−3.2 (3)	C9—C15—O16—C18	−170.39 (16)
C15—C9—C11—C12	178.28 (16)	C15—O16—C18—C19	90.5 (2)
C2—C1—C11—C9	−177.6 (2)	C15—O16—C18—C23	−94.2 (2)
C2—C1—C11—C12	0.4 (3)	C23—C18—C19—C20	−1.1 (3)
C14—N10—C12—C4	−173.36 (18)	O16—C18—C19—C20	173.85 (19)
C26—N10—C12—C4	6.0 (3)	C23—C18—C19—C24	179.2 (2)
C14—N10—C12—C11	5.4 (3)	O16—C18—C19—C24	−5.9 (3)
C26—N10—C12—C11	−175.25 (19)	C18—C19—C20—C21	0.0 (4)
C3—C4—C12—N10	−179.4 (2)	C24—C19—C20—C21	179.8 (3)
C3—C4—C12—C11	1.8 (3)	C19—C20—C21—C22	0.4 (4)
C9—C11—C12—N10	−2.2 (3)	C20—C21—C22—C23	0.1 (4)
C1—C11—C12—N10	179.66 (17)	C19—C18—C23—C22	1.5 (3)
C9—C11—C12—C4	176.56 (17)	O16—C18—C23—C22	−173.29 (17)
C1—C11—C12—C4	−1.5 (3)	C19—C18—C23—C25	−177.9 (2)
C11—C9—C13—C8	−175.30 (17)	O16—C18—C23—C25	7.3 (3)
C15—C9—C13—C8	3.2 (3)	C21—C22—C23—C18	−1.0 (4)
C11—C9—C13—C14	5.5 (3)	C21—C22—C23—C25	178.4 (2)
C15—C9—C13—C14	−175.99 (15)	O30—S27—C31—F33	−178.7 (2)
C7—C8—C13—C9	−179.25 (18)	O29—S27—C31—F33	61.1 (3)
C7—C8—C13—C14	0.0 (3)	O28—S27—C31—F33	−58.3 (3)
C12—N10—C14—C5	175.98 (17)	O30—S27—C31—F32	−56.7 (2)
C26—N10—C14—C5	−3.4 (3)	O29—S27—C31—F32	−177.0 (2)
C12—N10—C14—C13	−3.0 (3)	O28—S27—C31—F32	63.7 (2)
C26—N10—C14—C13	177.62 (19)	O30—S27—C31—F34	62.4 (2)
C6—C5—C14—N10	−178.31 (18)	O29—S27—C31—F34	−57.9 (2)
C6—C5—C14—C13	0.7 (3)	O28—S27—C31—F34	−177.18 (19)
C9—C13—C14—N10	−2.4 (2)		

### Hydrogen-bond geometry (Å, °)

**Table:**

Cg4 is the centroid of the C18–C23 ring.

**Table:**

*D*—H···*A*	*D*—H	H···*A*	*D*···*A*	*D*—H···*A*
C2—H2···O28^i^	0.93	2.51	3.224 (3)	134
C25—H25B···O30^ii^	0.96	2.57	3.525 (3)	176
C26—H26A···Cg4^iii^	0.96 (3)	2.86 (2)	3.774 (3)	158 (3)
C26—H26B···O29^iii^	0.96 (3)	2.56 (3)	3.369 (4)	142 (2)

Symmetry codes:  (i) *x*−1, *y*, *z*−1; (ii) *x*, *y*, *z*−1; (iii) *x*−1, *y*, *z*.

### Table 2 C–F···π interactions (Å,°).

*Cg*1 and *Cg*3 are the centroids of the C9/N10/C11–C14 and C5–C8/C13/C14 rings, respectively.

**Table:**

*X*	*I*	*J*	*I*···*J*	*X*···*J*	*X*–*I*···*J*
C31	F32	*Cg*1^iv^	3.655 (3)	4.490 (4)	121.5 (2)
C31	F32	*Cg*3^iv^	3.886 (3)	3.974 (4)	84.1 (2)
C31	F33	*Cg*3^iv^	3.746 (3)	3.974 (4)	90.4 (2)
C31	F34	*Cg*3^iv^	3.481 (2)	3.974 (4)	101.9 (2)

Symmetry code: (iv) –*x* + 1, –*y*, –*z* + 1.

### Table 3 π–π interactions (Å,°).

*Cg*1, *Cg*2 and *Cg*3 are the centroids of the C9/N10/C11–C14, C1–C4/C11/C12 and C5–C8/C13/C14 rings, respectively. Cg*I*···Cg*J* is the distance between ring centroids. The dihedral angle is that between the planes of the rings *I* and *J*. *CgI*_Perp is the perpendicular distance of *CgI* from ring *J*. *CgI*_Offset is the distance between *CgI* and perpendicular projection of *CgJ* on ring *I*.

**Table:**

*I*	*J*	*CgI*···*CgJ*	Dihedral angle	*CgI*_Perp	*CgI*_Offset
1	3^v^	3.502 (2)	2.71 (10)	3.473 (1)	0.445 (1)
2	3^v^	3.977 (2)	6.38 (11)	3.286 (1)	2.240 (1)
3	1^v^	3.502 (2)	2.71 (10)	3.470 (1)	0.480 (1)
3	2^v^	3.977 (2)	6.38 (11)	3.503 (1)	1.883 (1)

Symmetry code: (v) –*x* + 1, –*y* + 1, –*z* + 1.
